# Mr. Whittle's Observations on the Couching Needle

**Published:** 1806-02

**Authors:** Oldfeld Whittle

**Affiliations:** London


### *To the Editors of the Medical and Physical Journal.*

Gentlemen,

On perusing the last number of your Journal, I observed a communication from Mr. Simmons, in which he has made several observations on the division of the iris, some of which, appearing to me as rather singular, I have taken the liberty of addressing to you, a few remarks on the subject; and if you should esteem them worthy a place in your valuable miscellany, I shall consider myself much flattered by their insertion.

I am, &c.

*London, Jan.* 14, 1806. OLDFELD WHITTLE.

---

Mr. Simmons, (if we may be allowed to draw any inference from the bias of his practice, as well as from what he has written on this subject,) considers the couching needle, which was recommended to the profession by Mr. Hey, to be an instrument well adapted to the performance of this operation. Differing from Mr. S. in this respect, I shall beg leave to state my reasons for so doing, as well as to offer a few remarks which naturally arise out of that difference.

Mr. Simmons, says, " It is a maxim in surgery, to use as few instruments as possible, as well as to prefer those of the most simple structure." The propriety of this

 maxim, maxim, I trust no one will dispute, nor indeed is it in much danger of falling into disregard; for common sense will in general point out to us the futility and vanity of superfluous and unnecessary means, though it cannot at all times help us to those means which are necessary. I fear it will appear too evident in this case, that, in endeavouring to avoid the *perplexity of variety*, and in endeavouring to operate *efficiently, with simplicity and unity*, Mr. Simmons has been induced to lay aside those instruments which are necessary. In this light it has certainly appeared to me.

With the view of dividing the contracted iris, so as to extend the range of vision, Mr. S. says, "I pushed the needle into the eye behind the iris, with the flat side towards it, and passing the point through the pupil, I carried it forward anteriorly to the circumference of the iris next the inner canthus, when, turning the edges of the instrument horizontally, the iris on that side was completely divided by the inner edge, and with a sound distinctly audible." I felt not a little surprized at this part of the detail of the operation; for I supposed no one would risk the miscarriage of operation, so very delicate as those which belong to the eye, (and as far as regards their success,) so very hazardous, without being minutely acquainted with the necessary and exact form of their instruments. This, however, I am sorry to say, appears to be the case in the present instance, for Mr. Hey's needle, as it is delineated and described by himself, has no cutting edge whatever, except its small convex point; and having no cutting edge, none could be turned inwards to divide the iris. We must then conclude, either that Mr. Simmons was a little mistaken with regard to the configuration of needle, which, by the bye, would appear a plausible conclusion, when we consider the sound which the division of the iris produced: I say, we must conclude, either that it was not Mr. Hey's instrument which was used, or that Mr. Hey's intrument, most accommodating, assumed the necessary form and pressure of the time. But as this matter appears perfectly inexplicable without the assistance of Mr. Simmons, I must necessarily pass it by, and proceed.

Mr. S. says, that, "owing to a distortion in the eye itself, next the outer canthus, a very narrow border only of the iris was left; this I endeavoured to catch upon the point of the instrument; but it was now so loose as to endanger wounding the transparent cornea, near the centre of of it, in making the attempt." All this may be very easily conceived; and the probability of success may be also conceived, if we are aware, (as is assuredly the case) that the difficulty which would attend us in our attempts to shave the down from a floating feather, would also be equally an attendant on the vain endeavours to cut a suspended angle of the iris, when floating in the aqueous humour, by any single blade whatever. The couching needle is certainly an instrument, the use of which is easily acquired by any person; but we must not, on that account, attempt to perform operations with it, to which it is in no respect whatever adapted; particularly when we have other instruments, which are much more appropriate, though certainly of more difficult management.

Mr. Simmons seems to be aware of the great tendency which exists in the iris to close, after an artificial opening has been made through it. In what manner, I ask, would this tendency be most certainly obviated? I should conceive by cutting away a small portion of the iris; not merely by making a simple incision through it.

This Mr. Simmons seems to have been aware of, for he pushed his endeavours to this intent as far as was prudent. Now, instead of making use of the needle, had the cornea been divided from its union with the sclerotica, to the extent of about a fourth or a sixth part of its circumference, with a cornea knife, or with a lancet, every difficulty would have been removed. A pair of small scissars might then have been introduced, and by a proper application of their powers, we seldom fail to produce a proper effect. In case of their failure, however, there is sufficient room to introduce any other instrument that the case might seem to require; but if we begin the operation with the needle, we must also finish it with the needle: for we are utterly debarred the use of all other instruments, unless we make a fresh opening, which is certainly not to be recommended.

I trust then it will appear evident, that the couching needle is an instrument very unfit for the performance of this operation, more particularly Mr. Hey's, from its want of edges.

These are the principal of the observations which have occurred to me relating to the mere division of the iris; but before I conclude, I must beg leave to add a few others, on the propriety or impropriety of previously depressing the lens.

In

In most cases where the pupil is much contracted, or where it is totally obliterated, we in general find a greater or less degree of opacity in the lens, or its capsule, which, (after the successful performance of the operation, as far as relates to the division of the iris) still holds out an insurmountable barrier to the entrance of the rays of light. When this is the case, to the couching needle we must have recourse; but if we find strong adhæsions formed between the iris which had been divided, and the capsule of the lens, to what instrument then, must we fly? These adhæsions will be almost an inevitable effect of the operation, if the lens be suffered at the same time to retain its situation; besides, there is hardly any manner of instrument which we can employ to divide the iris, which will not at the same time injure the lens. If the scissars are used, one of the points will certainly wound it; if the needle, in its use, be pushed into the eye behind the iris in such manner that its point may be brought through the pupil, some part of the circumference of the lens, at least, must be pierced. The obvious and almost certain consequence of this, must be opacity, even where it did not pre-exist; the probable consequence, adhæsion. But we will suppose for a moment, that a portion of the iris might be removed, without the least injury being done to the lens or its capsule. What do we gain by the success of the operation? Nothing more than an indistinct, or double vision; for that portion of the rays of light which will pass through the lens, becomes properly refracted, and arrives at the retina in a focus; while those which will pass into the eye, beyond the limit of the circumference of the lens, will undergo but little change in their direction. Indistinct vision must therefore necessarily ensue, and this can only be remedied by the depression of the lens, which we have supposed to have escaped all injury, and to have retained its transparency.

It must therefore, I conceive, appear evident to every person who has paid attention to this subject, that, when the operation in question becomes necessary; it will be most prudent, first to depress the lens, and after the eye has again acquired its natural state, to make a sufficient opening into the jnnction of the cornea and sclerotica, with a small flat knife, and if the opening be sufficiently large, we shall experience but little difficulty in completely taking out a segment of the iris with a small pair of scissars. In this manner we do not run a risk of tearing the iris from its origin, in any part, which must be the case case when we trust to the needle. We have it in our power to remove a part of the iris; which is the best means of preventing its closure; and by this mode of operation, we may defy the power of an opaque lens or its adhæsions.

